# Downstream consequences of diagnostic error in pediatric anaphylaxis

**DOI:** 10.1186/s12887-018-1024-z

**Published:** 2018-02-07

**Authors:** H. Thomson, R. Seith, S. Craig

**Affiliations:** 1 School of Clinical Sciences at Monash Health, Monash University, Clayton, Australia; 2 Paediatric Emergency Department, Monash Children’s Hospital, Clayton, Australia; 3 Murdoch Children’s Research Institute, Parkville, Australia

**Keywords:** Anaphylaxis, Allergy, Diagnosis, Emergency department, Pediatrics

## Abstract

**Background:**

Pediatric anaphylaxis is commonly misdiagnosed in the Emergency Department (ED). We aimed to determine the impact of inaccurate diagnosis on the management and follow-up of pediatric anaphylaxis presenting to the ED.

**Methods:**

Retrospective chart review of ED management of children aged 0–18 years with allergic presentations to three EDs in Melbourne, Australia in 2014. Cases were included if an ED diagnosis of anaphylaxis was recorded, or the presentation met international consensus criteria for anaphylaxis.

**Results:**

Of the 60,143 pediatric ED presentations during the study period, 1551 allergy-related presentations were identified and reviewed. 187 met consensus criteria for anaphylaxis, and another 24 were diagnosed with anaphylaxis without meeting criteria. Of the 211 presentations, 105 cases were given an ED diagnosis of anaphylaxis and 106 cases were given an alternative diagnosis in ED.

Those diagnosed with anaphylaxis were more likely to receive epinephrine [85.7% vs 31.1% (OR = 13.27, 95% CI: 6.09–26.3)], to be observed for the recommended four hours [56.2% vs 29.2% (OR = 3.10, 95% CI 1.76–5.48, *p* < 0.001)], to have an epinephrine autoinjector available on discharge [81.9% vs 35.8% (OR = 4.12, 95% CI 2.07–8.22, *p* < 0.001)] and to be referred to an allergist [35.2% vs 16.0% (OR = 2.85, 95% CI 1.48–5.49, *p* < 0.01)]. Provision of anaphylaxis action plans and allergen avoidance advice was poorly documented for all patients.

**Conclusion:**

Accurate diagnosis of anaphylaxis in ED has a significant impact on observation times, prescription of epinephrine autoinjectors and referral to an allergist. These factors are key to reducing mortality and the significant morbidity that results from childhood anaphylaxis.

**Electronic supplementary material:**

The online version of this article (10.1186/s12887-018-1024-z) contains supplementary material, which is available to authorized users.

## Background

Anaphylaxis is a life threatening allergic reaction that occurs unexpectedly, often in young and otherwise healthy people. Anaphylaxis admission rates in Australia increased by 1.5 fold between 2005–2006 and 2011–2012, with the greatest acceleration in those aged 5 to 14 years [1].

While internationally accepted clinical criteria for anaphylaxis were established in 2005 [2], these criteria appear to be underused. International studies report that between 57-87% of patients meeting these criteria are given an alternative diagnosis [3–6].

Key management steps for anaphylaxis include early use of intramuscular epinephrine, a period of observation for potential biphasic reactions, prescription of an epinephrine autoinjector, and follow up with an allergist [2, 7, 8]. Despite this previous Australian [9–11] and international [3, 12, 13] studies have demonstrated that both immediate and long-term management of pediatric anaphylaxis appears suboptimal.

We have previously examined a cohort of children presenting to an Emergency Department (ED) with possible allergic disease and identified those meeting international consensus criteria for anaphylaxis. Approximately half of the children meeting these criteria did not receive a diagnosis of anaphylaxis during their ED presentation [14]. Multiple logistic regression identified the following factors associated with an ED diagnosis of anaphylaxis: previous anaphylaxis, arrival by ambulance, high-acuity triage category and presentation to a tertiary hospital. Resolution of symptoms and signs of at least one organ system prior to arrival was associated with reduced odds of an ED diagnosis of anaphylaxis (see Additional file 1: Table S1).

This paper aims to describe the impact of inaccurate ED diagnosis on both immediate and long-term management of pediatric anaphylaxis in the same cohort.

## Methods

A retrospective structured chart review was conducted of pediatric patients who presented to three hospitals in south east Melbourne, Australia with allergic symptoms in 2014. Our hospital network consists of a pediatric tertiary center with a dedicated pediatric ED, and two secondary hospitals with mixed adult / pediatric EDs.

Ethics approval was obtained from the Monash Health and Monash University Human Research Ethics Committees.

### Patient selection

An initial database was established from our institution’s ED electronic medical record (Symphony, version 2.29.3. Ascribe Ltd. Bolton, United Kingdom). Patients were included if there were under the age of 18 and had a recorded presenting complaint or final ED diagnosis that was compatible with anaphylaxis or an allergic reaction. Screened presentations included those with a specific triage presenting complaints included “allergic reaction” and “anaphylaxis” or ED diagnostic codes including “allergy”, “anaphylaxis”, “urticara”, “angioedema”, “food allergy”, “rash due to food”, “drug reaction”, “hay fever”, and “rash due to drug”. If a child had repeated visits, each was included as a separate presentation.

Patients were excluded if they left ED before treatment was completed, if they were transferred for observation from another hospital or clinical notes were missing.

#### Classification of anaphylaxis

Anaphylaxis was defined using criteria from the 2005 Second Symposium on the definition and management of anaphylaxis (Table 1) [2]. We defined hypoxemia as an oxygen saturation of less than 90% regardless of age or oxygen therapy.

**Table 1:** Anaphylaxis criteria (Adapted from: Sampson HA, Munoz-Furlong A, Campbell RL et al. Second symposium on the definition and management of anaphylaxis: summary report--second National Institute of Allergy and Infectious Disease/Food Allergy and Anaphylaxis Network symposium. Ann Emerg Med. 2006;47(4):373–80)

Anaphylaxis is highly likely when any one of the following 3 criteria are fulfilled
1. Acute onset of an illness (minutes to several hours) with involvement of the skin, mucosal tissue, or both (eg. Generalized hives, pruritus, or flushing, or swollen lips-tongue-uvula) and at least one of the following: a) Respiratory compromise (eg. Dyspnea, wheeze-bronchospasm, stridor, reduced PEF, hypoxemia) b) Reduced BP or associated symptoms of end-organ dysfunction (hypotonia [collapse], syncope, incontinence)
2. Two or more of the following that occur rapidly after exposure to a likely allergen for that patient (minutes to several hours): a) Involvement of the skin-mucosal tissue (eg. Generalized hives, itch-flush, swollen lips-tongue-uvula) b) Respiratory compromise (eg. Dyspnea, wheeze-bronchospasm, stridor, reduced PEF, hypoxemia) c) Reduced BP or associated symptoms (eg. Hypotonia [collapse] syncope, incontinence) d) Persistent gastrointestinal symptoms (eg. Crampy abdominal pain, vomiting)
3. Reduced BP after exposure to known allergen for that patient (minutes to several hours): a) < 3 months = < 60 mmHg b) 3–12 months = < 65 mmHg c) 1–4 years = < 70 mmHg d) 5–12 years = < 80 mmHg e) Over 12 years = < 95 mmHg

*PEF* Peak expiratory flow, *BP* blood pressure

Severity of anaphylaxis was graded using a scale proposed by Brown which defines a severe reaction as including hypoxia, hypotension or neurological compromise [15].

#### Data extraction and processing

A standard data extraction form was developed in consultation with emergency physicians and allergists within our institution, and was tested and modified prior to implementation in the study. The primary investigator who conducted the screening and data collection was not blinded to the study objective. Periodic meetings were held with the supervising emergency physician to discuss and gain consensus on any points of ambiguity.

The primary data sources were the ED medical record, including medical and nursing notes, medication and intravenous fluid charts, and observation charts. Additional information regarding pre-hospital care and clinical features was obtained from ambulance records.

#### Variables assessed

Variables to be assessed were based on previous studies of anaphylaxis [3, 9, 10, 13, 16–21]. These variables included age, sex, Australasian Triage Scale category, history of anaphylaxis and atopic diseases, details of anaphylactic events (allergen, exposure timing, location), vital signs, and clinical manifestations (including involvement of cutaneous, respiratory, gastrointestinal or cardiovascular systems). If more than one possible food trigger could be identified from the history, the eliciting agent was recorded as “mixed.” As “persistent gastrointestinal symptoms” are not explicitly defined in the diagnostic criteria used, we applied a threshold of 30 min. Details on ED management included medication administration, discharge prescriptions, outpatient referrals and documented discharge advice.

### Data analysis

Data entry and analysis were performed using SPSS Statistics (SPSS Statistics for Macintosh, Version 22.0; IBM Corp. Armonk, NY). Data queries were addressed by at least two investigators reviewing the original record. Descriptive statistics were reported as percentages for discrete variables. Continuous data was described as median with interquartile ranges (IQR). Differences between nonparametric continuous variables were determined using the Mann-Whitney U test. Differences between groups for categorical variables were determined using Fisher’s exact test for two by two tables and chi square test for larger contingency tables. *P* values < 0.05 were considered statistically significant.

## Results

**Fig. 1:**
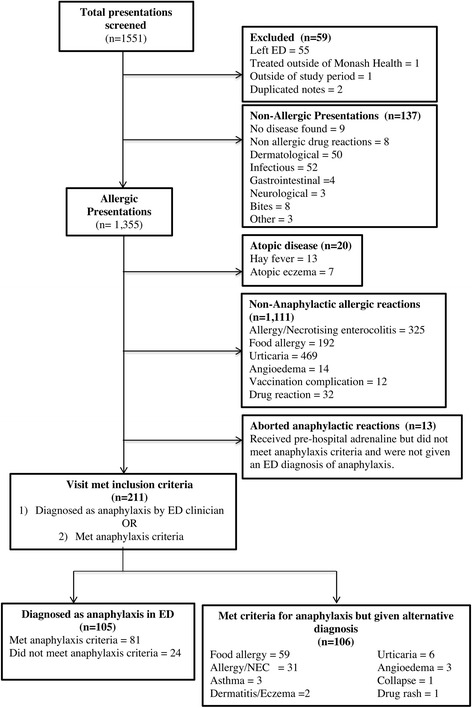
Study flow

During the study period there were 60,143 pediatric ED presentations across the three hospitals. 1551 clinical records were identified and reviewed (Fig. 1). Of these, 211 ED presentations were either diagnosed as anaphylaxis on ED discharge (105 patients, of whom 81 fulfilled anaphylaxis criteria and 24 did not), or met criteria for anaphylaxis but received an alternative diagnosis on ED discharge. Demographic and clinical features of these presentations are presented in Table 2.

**Table 2:** Baseline data and presentation of pediatric anaphylaxis patients

	Overall (*n* = 211) n(%)	ED Dx anaphylaxis (*n* = 105) n(%)	ED NOT Dx anaphylaxis (*n* = 106) n(%)	Odds ratio (95% CI)	*P* value
Demographics					
Age in years, median (IQR)	6 (2–11)	6 (3–11.5)	5 (2–10.3)	N/A	0.18
Boys, n (%)	131 (62.1)	64 (60.9)	67 (62.2)	N/A	0.74
Allergen class					
Food	173 (82.0)	91 (86.7)	82 (77.4)	N/A	0.35
Drugs	8 (3.8)	3 (2.9)	5 (4.7)		
Insect	2 (0.9)	1 (0.9)	1 (0.9)		
Other / unknown	28 (13.3)	10 (9.5)	18 (16.9)		
Organ system involved					
Cutaneous	203 (96.2)	97 (92.4)	105 (99.1)	0.12 (0.02–0.94)	0.02
Respiratory	159 (75.3)	70 (66.7)	89 (84.0)	0.38 (0.20–0.74)	< 0.01
Gastrointestinal	53 (25.1)	32 (30.5)	21 (19.8)	1.77 (0.94–3.34)	0.08
Cardiovascular	23 (10.9)	14 (13.3)	9 (8.5)	1.66 (0.68–4.02)	0.28
Neurological	27 (12.8)	19 (18.1)	8 (7.5)	2.71 (1.13–6.50)	0.02
Median (IQR) number of organ systems involved during episode	2 (2–2)	2 (2–2)	2 (2–3)	N/A	0.62
Median (IQR) number of organ systems involved at time of ED arrival	1 (0–1)	1 (0–1)	1 (0–2)	N/A	0.29
At least one organ system resolved prior to ED	157 (74.4)	68 (64.7)	89 (83.4)	0.35 (0.18–0.68)	0.001
All symptoms resolved prior to ED arrival	60 (28.4)	28 (26.7)	32 (30.2)	0.84 (0.45–1.54)	0.65
Severe anaphylaxis	28 (13.3)	20 (19.0)	8 (7.5)	2.88 (1.21–6.88)	0.02

### Biphasic reactions

There were seven biphasic reactions identified (3.3%). Three were diagnosed with anaphylaxis, three with food allergy and one with allergy. The second phase was anaphylactic in six of the reactions, while one of the reactions involved only respiratory features. The median time to biphasic reactions from resolution of initial symptoms was 115 min (IQR 68.5–144.5). In the initial reactions, three patients required epinephrine and three patients received steroids. Five of the second phase reactions required epinephrine.

### Management

Overall, epinephrine was administered during 123 presentations. Of these, 51 patients used an epinephrine autoinjector. Twenty patients had an autoinjector but did not use it. Twenty-two received epinephrine from ambulance paramedics, and the remainder received epinephrine in the ED. Most patients required a single dose of epinephrine, however, 16 received two doses, 3 three doses, and two patients received epinephrine infusions both after receiving two intramuscular doses of epinephrine.

Overall 81.5% of patients received an H1 antihistamine and 55.5% of patients received a steroid during their care. 58.3% received epinephrine at some point during their episode of anaphylaxis. Patients who were correctly diagnosed with anaphylaxis were more likely to receive epinephrine throughout their care and to receive multiple doses of epinephrine (Table 3).

**Table 3:** Management of pediatric anaphylaxis patients

	Overall (*n* = 211) n(%)	ED Dx anaphylaxis (*n* = 105) n(%)	ED NOT Dx anaphylaxis (*n* = 106) n(%)	Odds ratio (95% CI)	*P* value
Pre hospital					
Epinephrine Auto injector	51 (24.2)	37 (35.2)	14 (13.2)	3.58 (1.8–7.21)	< 0.001
Antihistamine	99 (46.9)	53 (50.5)	46 (43.4)	1.33 (0.78–2.28)	0.34
Steroid	22 (10.4)	13 (12.4)	9 (8.5)	1.52 (0.6–3.63)	0.38
Ambulance treatment (*n* = 106)					
Epinephrine	22 (20.8)	21/66 (31.8)	1/40 (2.5)	19.05 (3.0–202.6)	0.0001
Nebulized epinephrine	2 (1.9)	1/66 (1.5)	1/40 (2.5)	0.6 (0.03–11.67)	1.000
Oxygen	12/106 (11.3)	12/66 (18.2)	0/40 (0)	∞ (2.6 – ∞)	0.003
Beta agonist	14/106 (13.2)	12/66 (18.2)	2/40 (5)	4.38 (0.97–20.4)	0.074
Steroids	8/106 (7.5)	6/66 (9.1)	2/40 (5)	1.9 (0.44–9.59)	0.71
Fluids	3/106 (2.8)	3/66 (4.5)	0/40 (0)	∞ (0.52 - ∞)	0.29
ED treatment					
Epinephrine	48 (22.7%)	31 (29.5%)	17(16.0%)	2.20 (1.13–4.27)	0.02
Nebulized epinephrine	11 (5.2%)	8 (7.6%)	3 (2.8%)	2.8 (0.73–11.00)	0.13
Epinephrine infusion	2 (0.9%)	1 (1.0%)	1 (0.9%)	1.01 (0.06–16.37)	1
Steroid	97 (46.0%)	45 (42.9%)	52 (49.1%)	0.78 (0.45–1.34)	0.41
H1 antihistamine	100 (47.4%)	43 (41.0%)	57 (53.8%)	0.60 (0.35–1.03)	0.07
H2 Antihistamine (Ranitidine)	3 (1.4%)	3 (2.9%)	0 (0%)	7.27 (0.37–142.67)	0.12
Oxygen	9 (4.3%)	6 (5.7%)	3 (2.8%)	2.08 (0.51–8.55)	0.33
Inhaled beta agonist	17 (8.1%)	9 (8.6%)	8 (7.5%)	1.15 (0.43–3.10)	0.81
Intubation	1 (0.5%)	0 (0%)	1 (0.0%)	0.33 (0.01–8.28)	1
IV fluids 1st hour	7 (3.3%)	6 (5.7%)	1 (0.9%)	6.3 (0.75–53.82)	0.07
IV fluids in 24 h	8 (3.8%)	6 (5.7%)	2 (1.9%)	3.15 (0.62–15.99)	0.17
Overall management					
Epinephrine	123 (58.3%)	90 (85.7%)	33 (31.1%)	13.27 (6.69–26.3)	< 0.001
1 dose	104 (49.3%)	75 (71.4%)	29 (27.4%)	N/A	< 0.001
2 doses	16 (7.6%)	12 (11.4%)	4 (3.8%)		
3 doses	3 (1.4%)	3 (2.9%)	0 (0%)		
H1 Antihistamine	172 (81.5%)	84 (80.0%)	88 (83.0%)	0.82 (0.41–1.64)	0.60
Steroid	117 (55.5%)	57 (54.3%)	60 (56.6%)	0.91 (0.53–1.57)	0.78

Eighty-eight patients meeting diagnostic criteria for anaphylaxis did not receive epinephrine. Seventy three (83%) were not diagnosed as anaphylaxis by ED staff. Resolution of symptoms had occurred prior to ED arrival in 73 (83%) of these. A minority were administered other treatment, including inhaled beta-agonists (4 patients), nebulized epinephrine (1 patient) and oral corticosteroids (1 patient).

### Observation and discharge

Patients diagnosed with anaphylaxis were far more likely to be observed in ED for more than four hours. In those given epinephrine, those given a diagnosis of anaphylaxis were far more likely to be observed for four hours after their epinephrine dose. Patients diagnosed with anaphylaxis were more likely to be admitted to short stay or ICU than patients given an alternative diagnosis. Those not diagnosed with anaphylaxis were more likely to be directly discharged home (Table 4).

**Table 4:** ED disposition and discharge arrangements

	Overall (*n* = 211) n(%)	ED Dx anaphylaxis (*n* = 105) n(%)	ED NOT Dx anaphylaxis (*n* = 106) n(%)	Odds ratio (95% CI)	*P* value
Action plan					
Action Plan provided	59 (28.0%)	34 (32.4%)	25 (23.6%)	N/A	0.12
Already had action plan	5 (2.4%)	4 (3.8%)	1 (0.9%)		
No mention of action plan	147 (69.7%)	67 (63.8%)	80 (75.5%)		
Allergen avoidance advice provided	45 (21.3%)	23 (21.9%)	22 (20.8%)	1.07 (0.55–2.07)	0.87
Autoinjector prescription					
Autoinjector available on discharge	124 (58.8%)	86 (81.9%)	38 (35.8%)	4.12 (2.07–8.22)	< 0.001
Autoinjector prescribed	109 (51.7%)	76 (72.4%)	33 (31.1%)	N/A	< 0.001
Already had autoinjector	15 (7.1%)	10 (9.5%)	5 (4.7%)		
No mention of autoinjector in notes	87 (41.2%)	19 (18.1%)	68 (64.2%)		
Allergist referral					
Allergist referral made	54 (25.6%)	37 (35.2%)	17 (16.0%)	2.85 (1.48–5.49)	< 0.01
Allergist recommended but no referral	55 (26.1%)	26 (24.8%)	29 (27.4%)	0.87 (0.47–1.62)	0.75
Known to allergist^a^	46 (21.8%)	33 (31.4%)	13 (12.3%)	3.28 (1.61–6.68)	< 0.001
Mention of allergist in ED notes	126 (59.8%)	74 (70.5%)	52 (49.1%)	2.48 (1.41–4.37)	< 0.01
Observation					
Median length of stay in minutes (IQR)	N/A	265 (201.5–372)	181.5 (117–272.25)	N/A	< 0.001
In ED > 4 h	90 (42.7%)	59 (56.2%)	31 (29.2%)	3.10 (1.76–5.48)	< 0.001
Median time of observation post epinephrine in minutes(IQR)	314 (256–629)	216 (129–490.5)	N/A	N/A	0.01
Observed > 4 h post epinephrine	28 (90.3%)	6 (35.3%)	17.11 (3.63–80.77)		< 0.001
ED disposition					
Discharged home	57 (27.0%)	11 (10.5%)	46 (43.4%)	N/A	< 0.001
Short stay	132 (62.6%)	83 (79.0%)	49 (46.2%)		
Ward	20 (9.5%)	9 (8.6%)	11 (10.4%)		
ICU Transfer	2 (0.9%)	2 (1.9%)	0 (0%)		

^a^Not all patients known to an allergist were referred to see them or recommended to see the again

### Discharge advice

There was no significant difference in the discharge advice given to both groups. Overall 28% of patients received an anaphylaxis action plan while 2.4% already had a plan in place. 21% of patients were give allergen avoidance advice (Table 4).

Patients who were correctly diagnosed with anaphylaxis were far more likely to be prescribed an epinephrine autoinjector or to already have one (Table 4). Of the 109 patients given an autoinjector prescription, 62 (57%) had documentation of education on how to use the device. Diagnosis did not impact on autoinjector education.

Patients who were correctly diagnosed with anaphylaxis were more likely to be referred to see an allergist compared to those who received an alternative diagnosis (Table 4).

## Discussion

To our knowledge, this is the largest cohort of pediatric anaphylaxis patients identified in Australia and one of the largest internationally within a one-year period. It is notable that a number of children meeting clinical criteria for anaphylaxis had resolution of at least some of their symptoms prior to ED arrival.

In our cohort 58.3% of anaphylaxis patients received epinephrine during their care. This is consistent with previously reported rates of 19–94% [3, 6, 9, 10, 18, 22]. In our cohort ED diagnosis had a significant impact on epinephrine administration. 85.7% of patients diagnosed correctly with anaphylaxis received epinephrine compared to 31.1% of patients given an alternative diagnosis. This is surprising, as if a reaction was considered severe enough to require epinephrine it would follow that it would be diagnosed as anaphylaxis.

The majority of patients who did not receive epinephrine had resolution of symptoms by the time they arrived to the ED. In contrast De Silva et al. reported that in the 24% of patients who did not receive epinephrine in their study, the vast majority had indications for epinephrine (*n* = 22/29, 76%), while six had resolution of symptoms (21%) and one parent refused [9]. However, the authors did not mention the specific indications for epinephrine. It remains unclear if all patients require epinephrine, particularly if symptoms are resolving rapidly.

Of the 106 patients who arrived by ambulance only 21% received epinephrine from paramedics. Paramedic epinephrine administration is safe [23]. Ambulance Victoria guidelines emphasise a low threshold for administering epinephrine to all anaphylaxis cases [24]. Despite this a UK study of 816 anaphylaxis ambulance cases found that only 14% received epinephrine [25]. It may be that patients did not meet the criteria for anaphylaxis until they arrived to the emergency department or that paramedics opted to transport the patient and for the decision for epinephrine to be made by the ED clinician. Similarly, 20 of the 71 patients who had an autoinjector available did not use it. This rate of 28% of patients not using their autoinjector was significantly lower than the 71% reported in 86 Australian children with recurrent anaphylaxis [26]. In order to ensure the early administration of epinephrine pre hospital providers, patients and their carers must be educated in its indications.

While delays in epinephrine administration result in increased hospitalization and fatalities [27], anaphylactic reactions can naturally resolve without epinephrine [7]. There are no guidelines recommending that anaphylaxis patients who are no longer symptomatic should receive epinephrine. It may be reasonable to withhold epinephrine in asymptomatic patients as long as they are appropriately observed and followed up due to the risks of biphasic and recurrent reactions.

In our cohort many cases of anaphylaxis were treated with less effective medications. 78 (37.0%) patients received pharmacological treatment without epinephrine administration. This included 44 (20.8%) patients receiving steroids and 75(35.5%) patients receiving an antihistamine without also being given epinephrine. Overall 55.5% of patients received steroids and 81.5% of patients received an H1 antihistamine at some point in their care. These results are similar to those found in the Australian literature with steroid rates reported between 44-77% an antihistamine use reported as between 44-88% in pediatric anaphylaxis [9–11, 22]. Australian guidelines acknowledge that while steroid use is common, their benefits in anaphylaxis is unproven and that antihistamines have no role in acute management of anaphylaxis [8].

There were seven biphasic reactions recorded in our study, making up 3% of all reactions. This is at the low end of the 3–14.7% [6, 16, 18, 28–31] reported in the literature. The biphasic reactions identified in our cohort were clinically significant as five required epinephrine while one patient was admitted to ICU.

Biphasic reactions can occur between 1 to 72 h after the resolution of initial symptoms [32]. In our cohort the median time to biphasic reactions was 1.92 h (IQR 1.14–2.41). There was one outlier with a reaction occurring after 10.2 h. The median time in our small cohort is significantly less than the 4.7 h [16] (IQR 3.3–7) reported by Mehr et al. and 8.8 h [29] (range 3–20.5) reported by Alqurashi et al., although these studies included follow-up of patients after hospital discharge.

Australian guidelines recommend four hours of observation after the last dose of epinephrine [8]. In our cohort of the 31 children given epinephrine in ED and diagnosed with anaphylaxis, 90.3% were observed for four hours after their last epinephrine dose. Conversely, only 6 of the 17 (35.3%) who received epinephrine with an alternative diagnosis were observed for more than four hours. The reasons for shorter observation times after epinephrine administration in these patients is unclear.

Overall only 42.7% of patients were observed for more than four hours in ED. This is less than the 64% reported by Murad et al. in an Australian ED [11]. Being correctly diagnosed with anaphylaxis significantly increased the chance a child was observed for more than four hours (56.2% vs 29.2%, *p* < 0.001). International recommendations for observation vary from 4 to 24 h, while some suggest admitting any patient with anaphylaxis [2, 7, 33]. Previous studies have reported that only 2–6% of children with biphasic reactions require intervention [28, 29]. However being in a setting that can provide immediate care is potentially lifesaving.

As it is difficult to predict which children with experience biphasic reactions, guidelines continue to recommend at least four hours of observation, and a low threshold for admission. Patients and their parents must also receive appropriate education, action plans and an epinephrine autoinjector before leaving hospital to reduce the frequency and severity of future reactions [7].

In our cohort only 28% of patients were documented to receive a written anaphylaxis action plan, while 2.4% already had one. Only 21.3% had documentation of being given allergen avoidance advice. This is similar to the 35% reported by Rudders et al. [4] Correct anaphylaxis diagnosis did not impact on the advice given. The importance of such advice is highlighted by the fact that 26.1% of anaphylaxis patients in our cohort had a known allergy to the causative agent. This is consistent with previously reported rates of 14–50% [4, 10, 13, 19, 31].

In our cohort 51.7% of overall anaphylaxis presentations were prescribed an epinephrine autoinjector, while 7.1% already had one available. This is similar to the previous reported rates of 17.5–63.0% [3, 4, 6, 10, 13, 19, 22, 31]. Correct anaphylaxis diagnosis had a profound impact on autoinjector prescription. 81.9% of those diagnosed correctly as anaphylaxis went home with an autoinjector compared to 35.8% of those given an alternative diagnosis. Of the 109 patients give an autoinjector prescription in our cohort, 47 (41.1%) received no documented education on how to use the device. This was not influenced by correct anaphylaxis diagnosis.

One proposed method to improve anaphylaxis diagnosis and management is the introduction of departmental protocols. In two separate Spanish and American centres there were significant improvements in epinephrine administration, observation, admission rates, allergist referral, and discharge instructions following the introduction of an anaphylaxis protocol [20, 34]. The US study also saw an increase in anaphylaxis cases diagnosed after the implementation of the protocol. This was suggested to be due to increased awareness [34].

The key to long-term care is referral to an allergist as they can verify triggers, provide education and offer immunotherapy [7]. Despite this, previous studies have reported referral rates between 24 and 71% [4, 6, 10–13, 19, 22]. In our cohort only 25.6% of overall anaphylaxis patients were referred to an allergist. 70.5% of patients correctly diagnosed with anaphylaxis were known to an allergist, referred or recommended to see one compared to 49.1% of those given an alternative diagnosis. Our health service does not have a public pediatric allergy service; the only such service is situated in the state’s only other tertiary pediatric hospital. However, there are also a number of private pediatric allergists in Melbourne. The low rate of documented allergist referrals may be due to patients being advised to seek a referral through their general practitioner, although it was unclear whether this was the case.

Anaphylaxis remains a difficult clinical diagnosis based on pattern recognition. Sargant et al. proposed that anaphylaxis can masquerade as severe asthma [35], in our cohort only three patients identified as meeting anaphylaxis criteria were diagnosed with asthma. Cutaneous features are absent in up to 20% of presentations [10] or may be masked by antihistamines [36]. Similarly the lack of previous reactions and allergies cannot be relied upon to rule out anaphylaxis. We have previously published that in our cohort only 31% of patients presenting with anaphylaxis had a previous anaphylactic reaction. While this is more that the 17% reported by De Silva et al. [9], this highlights that the vast majority of paediatric anaphylactic presentations are first presentations.

The primary limitation of this study is the retrospective design and reliance on complete documentation in the clinical records. Additionally, we do not have a pediatric allergy service in our hospital network, so we were unable to obtain the opinion of a specialist pediatric allergist on each medical record. However, in keeping with recommended practice for chart review studies [37], our data points were clearly defined, and a standardized data extraction form was used to improve accuracy and consistency of data collection. We applied diagnostic criteria from established guidelines on the diagnosis of anaphylaxis to each patient’s data, ensuring uniform interpretation of recorded clinical features.

## Conclusion

In summary, in our cohort of over 200 cases, pediatric anaphylaxis appears to be under diagnosed. This is associated with shorter observation times, less autoinjector prescriptions and less allergist referrals. Patients also rarely receive anaphylaxis action plans or allergen avoidance advice. This places children at significant unnecessary risk of both biphasic and recurrent reactions.

As EDs are the treatment setting for most anaphylaxis visits this represents an important opportunity to improve patient care.

## Additional file

Additional file 1: Predictors of emergency department diagnosis of pediatric anaphylaxis (after multiple logistic regression). This Table outlines factors that were associated with an increased rate of anaphylaxis diagnosis. (DOCX 11 kb)
Additional file 2: Study data set. (XLSX 116 kb)

## Abbreviations

ED: Emergency Department
IQR: Inter quartile range

## References

[1] Mullins RJ, Dear KB, Tang ML (2015). Time trends in Australian hospital anaphylaxis admissions in 1998-1999 to 2011-2012. J Allergy Clin Immunol.

[2] Sampson HA, Munoz-Furlong A, Campbell RL, Adkinson NF, Bock SA, Branum A (2006). Second symposium on the definition and management of anaphylaxis: summary report--second National Institute of allergy and infectious disease/Food Allergy and Anaphylaxis Network symposium. Ann Emerg Med.

[3] Huang F, Chawla K, Jarvinen KM, Nowak-Wegrzyn A (2012). Anaphylaxis in a new York City pediatric emergency department: triggers, treatments, and outcomes. J Allergy Clin Immunol.

[4] Rudders SA, Banerji A, Corel B, Clark S, Camargo CA (2010). Multicenter study of repeat epinephrine treatments for food-related anaphylaxis. Pediatrics.

[5] Ross MP, Ferguson M, Street D, Klontz K, Schroeder T, Luccioli S (2008). Analysis of food-allergic and anaphylactic events in the National Electronic Injury Surveillance System. J Allergy Clin Immunol.

[6] Sidhu N, Jones S, Perry T, Thompson T, Storm E, Melguizo Castro MS (2016). Evaluation of anaphylaxis Management in a Pediatric Emergency Department. Pediatr Emerg Care.

[7] Simons FE, Ebisawa M, Sanchez-Borges M, Thong BY, Worm M, Tanno LK (2015). 2015 update of the evidence base: world allergy organization anaphylaxis guidelines. World Allergy Organ J.

[8] ASCIA (2016). Acute management of anaphylaxis guidelines.

[9] de Silva IL, Mehr SS, Tey D, Tang ML (2008). Paediatric anaphylaxis: a 5 year retrospective review. Allergy.

[10] Braganza SC, Acworth JP, McKinnon DR, Peake JE, Brown AF (2006). Paediatric emergency department anaphylaxis: different patterns from adults. Arch Dis Child.

[11] Murad A, Katelaris CH (2016). Anaphylaxis audit in a busy metropolitan emergency department: a review of real life management compared to best practice. Asia Pac Allergy.

[12] Vetander M, Helander D, Flodstrom C, Ostblom E, Alfven T, Ly DH (2012). Anaphylaxis and reactions to foods in children--a population-based case study of emergency department visits. Clin Exp Allergy.

[13] Russell S, Monroe K, Losek JD (2010). Anaphylaxis management in the pediatric emergency department: opportunities for improvement. Pediatr Emerg Care.

[14] Thomson H, Seith R, Craig S. Inaccurate diagnosis of paediatric anaphylaxis in three Australian emergency departments. J Paediatr Child Health. 2017;53(7):698–704. 10.1111/jpc.13483 28670809

[15] Brown SG (2004). Clinical features and severity grading of anaphylaxis. J Allergy Clin Immunol.

[16] Alqurashi W, Stiell I, Chan K, Neto G, Alsadoon A, Wells G. Epidemiology and clinical predictors of biphasic reactions in children with anaphylaxis. Ann Allergy Asthma Immunol. 2015;115(3):217–23. 10.1016/j.anai.2015.05.013 26112147

[17] Silva R, Gomes E, Cunha L, Falcao H (2012). Anaphylaxis in children: a nine years retrospective study (2001-2009). Allergol Immunopathol (Madr).

[18] De Swert LF, Bullens D, Raes M, Dermaux AM (2008). Anaphylaxis in referred pediatric patients: demographic and clinical features, triggers, and therapeutic approach. Eur J Pediatr.

[19] Mehl A, Wahn U, Niggemann B (2005). Anaphylactic reactions in children--a questionnaire-based survey in Germany. Allergy.

[20] Arroabarren E, Lasa EM, Olaciregui I, Sarasqueta C, Munoz JA, Perez-Yarza EG (2011). Improving anaphylaxis management in a pediatric emergency department. Pediatr Allergy Immunol.

[21] Dibs SD, Baker MD (1997). Anaphylaxis in children: a 5-year experience. Pediatrics.

[22] Nogic C, Belousoff J, Krieser D (2016). The diagnosis and management of children presenting with anaphylaxis to a metropolitan emergency department: a 2-year retrospective case series. J Paediatr Child Health.

[23] Manivannan V, Hyde RJ, Hankins DG, Bellolio MF, Fedko MG, Decker WW (2014). Epinephrine use and outcomes in anaphylaxis patients transported by emergency medical services. Am J Emerg Med.

[24] Victoria A. Clinical Practice Guidelines Doncaster. Victoria: Ambulance Victoria; 2016. [cited 2017 June 5]. Available from: http://www.ambulance.vic.gov.au/paramedics/clinical-practice-guidelines/

[25] Capps JA, Sharma V, Arkwright PD (2010). Prevalence, outcome and pre-hospital management of anaphylaxis by first aiders and paramedical ambulance staff in Manchester, UK. Resuscitation.

[26] Gold MS, Sainsbury R (2000). First aid anaphylaxis management in children who were prescribed an epinephrine autoinjector device (EpiPen). J Allergy Clin Immunol.

[27] Pumphrey RS (2000). Lessons for management of anaphylaxis from a study of fatal reactions. Clin Exp Allergy.

[28] Lee JM, Greenes DS (2000). Biphasic anaphylactic reactions in pediatrics. Pediatrics.

[29] Mehr S, Liew WK, Tey D, Tang ML (2009). Clinical predictors for biphasic reactions in children presenting with anaphylaxis. Clin Exp Allergy.

[30] Lertnawapan R, Maek-a-nantawat W (2011). Anaphylaxis and biphasic phase in Thailand: 4-year observation. Allergol Int.

[31] Manuyakorn W, Benjaponpitak S, Kamchaisatian W, Vilaiyuk S, Sasisakulporn C, Jotikasthira W (2015). Pediatric anaphylaxis: triggers, clinical features, and treatment in a tertiary-care hospital. Asian Pac J Allergy Immunol.

[32] Lieberman P (2005). Biphasic anaphylactic reactions. Ann Allergy Asthma Immunol.

[33] Muraro A, Roberts G, Worm M, Bilo MB, Brockow K, Fernandez Rivas M (2014). Anaphylaxis: guidelines from the European academy of allergy and clinical immunology. Allergy.

[34] Desai SH, Jeong K, Kattan JD, Lieberman R, Wisniewski S, Green TD (2015). Anaphylaxis management before and after implementation of guidelines in the pediatric emergency department. J Allergy Clin Immunol Pract.

[35] Sargant N, Erlewyn-Lajeunesse M, Benger J (2015). Does anaphylaxis masquerade as asthma in children?. Emerg Med J.

[36] Runge JW, Martinez JC, Caravati EM, Williamson SG, Hartsell SC (1992). Histamine antagonists in the treatment of acute allergic reactions. Ann Emerg Med.

[37] Kaji AH, Schriger D, Green S (2014). Looking through the retrospectoscope: reducing bias in emergency medicine chart review studies. Ann Emerg Med.

