# Supplementary material for: Downstream consequences of diagnostic error in pediatric anaphylaxis
Source: BMC Pediatr. 2018 Feb 7;18:40. doi: 10.1186/s12887-018-1024-z (PMC5803891; doi:10.1186/s12887-018-1024-z)
Supplement: Additional file 1: — Predictors of emergency department diagnosis of pediatric anaphylaxis (after multiple logistic regression). This Table outlines factors that were associated with an increased rate of anaphylaxis diagnosis. (DOCX 11 kb) [file 12887_2018_1024_MOESM1_ESM.docx]

**Additional file 1: Table S1** Predictors of emergency department diagnosis of pediatric anaphylaxis (after multiple logistic regression)

| Predictor | Odds ratio (95% CI) | P value |
| --- | --- | --- |
| Previous anaphylaxis | 3.20 (1.52 – 6.75) | 0.002 |
| Australasian Triage Scale category 1 or 2 | 4.51 (2.20 – 9.25) | <0.001 |
| Arrival by ambulance | 2.80 (1.36 – 5.74) | 0.003 |
| Attendance to a tertiary hospital | 2.86 (1.44 – 5.67) | 0.003 |
| Resolution of signs/symptoms of involvement of at least one organ system | 0.27 (0.12 – 0.62) | 0.002 |
